# Evaluation of Haptic Feedback on Bimanually Teleoperated Laparoscopy for Endometriosis Surgery

**DOI:** 10.1109/TBME.2018.2870542

**Published:** 2018-09-20

**Authors:** Sergio Portolés Díez, Gianni Borghesan, Luc Joyeux, Christel Meuleman, Jan Deprest, Danail Stoyanov, Sebastien Ourselin, Tom Vercauteren, Dominiek Reynaerts, Emmanuel B. Vander Poorten

**Affiliations:** 1 Department of Mechanical EngineeringKU Leuven 169074 Leuven3001Belgium; 2 Department of Mechanical EngineeringKU Leuven 169074; 3 Department of Development and RegenerationUniversitaire Ziekenhuizen Leuven 82306; 4 Department of Development and RegenerationUniversitaire Ziekenhuizen Leuven 82306; 5 Wellcome/EPSRC Centre for Interventional and Surgical SciencesUniversity College London 15658; 6 Wellcome/EPSRC Centre for Interventional and Surgical SciencesUniversity College London 15658

**Keywords:** Surgical robotics, haptics, force-feedback, endometriosis, minimal invasive surgery, laser surgery, surgical training

## Abstract

Robotic minimal invasive surgery is gaining acceptance in surgical care. In contrast with the appreciated three-dimensional vision and enhanced dexterity, haptic feedback is not offered. For this reason, robotics is not considered beneficial for delicate interventions such as the endometriosis. Overall, haptic feedback remains debatable and yet unproven except for some simple scenarios such as fundamentals of laparoscopic surgery exercises. *Objective:* This work investigates the benefits of haptic feedback on more complex surgical gestures, manipulating delicate tissue through coordination between multiple instruments. *Methods:* A new training exercise, “endometriosis surgery exercise” (ESE) has been devised approximating the setting for monocular robotic endometriosis treatment. A bimanual bilateral teleoperation setup was designed for laparoscopic laser surgery. Haptic guidance and haptic feedback are, respectively, offered to the operator. User experiments have been conducted to assess the validity of ESE and examine possible advantages of haptic technology during execution of bimanual surgery. *Results:* Content and face validity of ESE were established by participating surgeons. Surgeons suggested ESE also as a mean to train lasering skills, and interaction forces on endometriotic tissue were found to be significantly lower when a bilateral controller is used. Collisions between instruments and the environment were less frequent and so were situations marked as potentially dangerous. *Conclusion:* This study provides some promising results suggesting that haptics may offer a distinct advantage in complex robotic interventions were fragile tissue is manipulated. *Significance:* Patients need to know whether it should be incorporated. Improved understanding of the value of haptics is important as current commercial surgical robots are widely used but do not offer haptics.

## I. Introduction

Robotic minimally invasive surgery (RMIS) is taking in an increasingly important role into modern surgery. In gynaecology, tubal reanastomosis or myomectomy are good candidates for RMIS. Surgeons greatly appreciate the way how complex tools can now be handled in an intuitive manner [1]. Laparoscopic instruments with distal Degrees of Freedom (DoFs) can be commanded by natural wrist and finger movements. Despite these advantages, there is still a significant learning curve associated to RMIS [2]. A common concern - partially explaining the long learning curve - is the absence of haptic feedback in present RMIS systems [3]–[6]. Surgeons, completely shield from physical interaction with the patient, must rely on visual cues, knowledge of the patient's anatomy and the own experience to correctly gauge the level of the governing interaction forces [7]. The absence of haptics leads to a long and costly learning process [2]. Humans can get accustomed to the absence of haptic feedback and, in fact, some highly experienced robotic surgeons do argue that haptics is not really needed for RMIS, if a proper stereovision system is available. Nevertheless, it is hypothesized that even such highly skilled surgeons are slowed down by requiring to visually estimate interaction forces. This additional mental load occasionally leads to inadequate surgical gestures [8], [9].

While current commercially available systems only foresee unilateral controllers, i.e., without haptic feedback, several commercial initiatives are said to be developing bilateral, i.e., including haptic feedback, platforms. Systems such as ALF-X by TransEnterix Inc. (Morrisvill, North Carolina, US) and SPORT by Titan Medical Inc. (Toronto, Canada) also the MIRO [10] recently licensed by Medtronic (Dublin, Ireland) include haptic technology. It can be expected that once a first competing system, with or without haptic feedback, enters the market, the inclination to include haptics in the current portfolio will be much higher, but at this point there does not seem yet a sufficient urgency for the existing companies to provide such technology. Aside from these commercial developments, many bilateral RMIS prototypes have been described in literature. A comprehensive overview of these developments can be found in [11] and [12]. Developments with good potential for clinical translation are devices that build on top of existing commercial systems. For example, a vibrotactile display added on top of an Intuitive Surgical Da Vinci S system was built by McMahan *et al.* [13] inspired by the bench-top system proposed earlier by Kontarinis *et al.* [14]. Koehn and Kuchenbecker demonstrated that both surgeons and non-surgeons preferred vibrotactile feedback above auditory cues on this system [15]. Pacchierotti [16] *et al.* used the BioTac sensor to gather tactile information from a tissue phantom. Haptic feedback was provided through two tactile channels mounted on a DaVinci. This system improved several performance metrics such as completion time and pressure exerted. Also other research studies [2], [12], [17]–[24] showed the potential value of haptic feedback, be it in fairly simple scenarios.

Tholey *et al.* showed that through fusion of visual and haptic feedback one can better characterize the stiffness of tissue. [2]. From a peg transfer experiment executed on a modified da Vinci system, King *et al.* [19] demonstrated that with tactile feedback, grasping forces are lower in RMIS. Deml *et al.* [20] studied the difference in terms of speed and accuracy between classic endoscopy and haptic enabled RMIS and the relation between the skills used by dissecting tissue model made on modelling material and cellular rubber. They concluded that haptic enabled RMIS is helpful to avoid trauma, however the amount of dissected surface decreased compared to manual intervention. Wagner *et al.* [17] investigated the role of haptics through analysis of blunt dissection on a clay model. A clear benefit in terms of instrument positioning accuracy was found in this research. A later study included an exercise for cannula insertion. The cannula was modelled as two pipes made from PVC and rubber. This experiment proved that force feedback reduced the applied force levels, but negatively affected the execution time of less experienced surgeons [25].

Knot tying considered as a basic RMIS skill forms a bit more challenging and representative task. Bethea *et al.* showed that already by simply visually displaying the force (not even feeding it back haptically), operators already apply more consistent and precise tension during knot tying [18]. A broader survey of the impact of haptics on RMIS is described by van der Meyden and Enayati *et al.* [12], [22]. They point at the growing evidence in favour of haptics in many critical aspects of surgery.

However a proof that haptics is absolutely necessary is still missing and further evidence e.g., through more involved experiments is needed to convince the clinical community. The majority of studies arguing in favour of haptics are limited to rather simple scenarios where only basic RMIS skills are investigated. More complex scenarios and models such as those employed by Wottawa *et al.* [24] who uses an animal model and investigates grasping force, are scarce. More sophisticated experiments where procedural skills are being evaluated on physical models are yet to be reported. Such studies might convince expert surgeons that earlier evidence demonstrating the benefit of haptics would likely transfer towards the Operating Room (OR).

This paper investigates the value of haptics in a more complex scenario, namely on a specially developed model for training surgery for treatment of endometriosis [26]. Endometriosis is typically treated endoscopically. By handling multiple instruments in a coordinated fashion, tissue that grows outside of the uterus is removed. Through careful excision, surgeons try to maximally spare the ovaries and the uterus. Such excisions can be made through laser ablation. A forceps, the second instrument, is used to properly position and tension the tissue were incisions are needed. Surgeons indicate to prefer laparoscopic over robotic treatment for this procedure. They argue that accurate force control to tension tissue is vital for this particular procedure and that it is impossible to reliably conduct the procedure when omitting haptics.

This paper proposes a dedicated training task for treatment of endometriosis, the Endometriosis Surgery Exercise (ESE). This new exercise will serve as an aid to investigate the value of haptics in safety-critical bimanual manipulation tasks. The paper further introduces a bimanual teleoperation setup consisting out of a PHANToM Premium 1.5 [27] from 3D Systemes (formerly Sensable) and a LoTESS [28] robot, both haptic masters that serve as input devices of the bimanual teleoperation setup. A modified LoTESS robot and Vesalius [29], an RCM robot controlling a laser laparoscope, form the two slave robots of the teleoperation system. All components are set up to conduct ESE experiments. A bilateral controller is installed between both masters and slaves. The controller relies on a combination of externally positioned force sensors to estimate the applied interaction force for the robot controlling the forceps (the laser robot is not considered as it is not supposed to contact tissue). The extracorporeal force measurement system has been earlier described by Willaert *et al.* [30]. A novel grasper was developed for manipulating tissue. User experiments consisting of three repetitions of the ESE where conducted with the novel system. All experiments were executed both with and without the haptic bilateral controller in random order. A detailed analysis of the effect of haptic feedback is conducted.

**Fig. 1.**
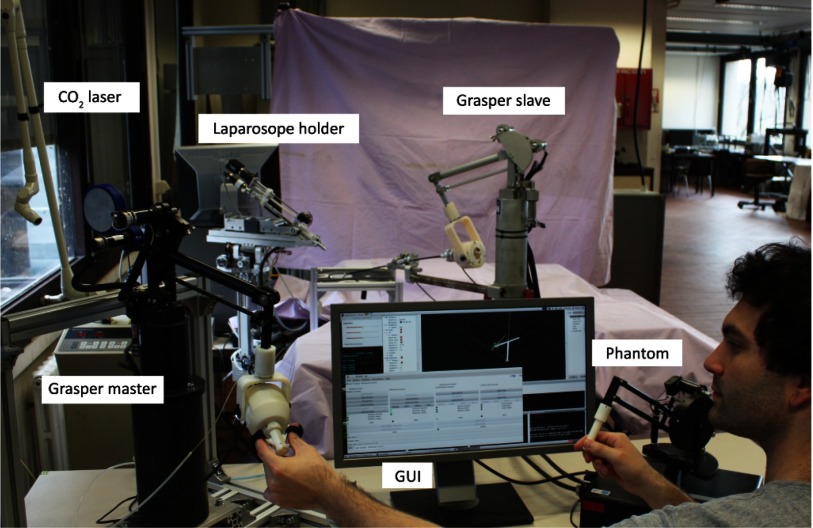
Overview of experimental bimanual laser surgery setup. Two master joysticks are controlled by the operator. Steering commands are sent to a laser laparoscope holding slave robot and a robot controlling a grasper. The interaction force exerted by the slave is fed back via a bilateral controller and is well perceivable by the operator.

The structure of this paper is as follows. Section II introduces the medical workflow of endometriosis surgery. This section also introduces the ESE as a novel model for RMIS training. In Section III the bimanual bilaterally teleoperated robotic platform for endometriosis surgery, seen in Fig. 1, is described in the necessary level of detail. The specificities of the experimental campaign that was launched are clarified in Section IV. This section introduces the results and the different metrics that were computed. Section V discusses the statistical significance of the obtained results. Subjective feedback of participants on the conducted experiments are collected here as well. Finally, a summary listing the main conclusions is included in Section VI.

## II. Endometriosis Surgery Exercise

This section introduces the ESE that was designed to train and validate robotic endometriosis treatment. After describing in greater detail the clinical background (Section II-A) and the current gold standard for treatment (Section II-B), the proposed training model is introduced in Section II-C.

### A. Clinical Background

Endometriosis is a gynaecological condition that involves endometriotic tissue. The tissue grows abnormally; reaching outside the uterus it expands and attaches to the peritoneum. Estimated prevalence of the condition is 10–15% of the women in childbearing age with highest incidence on 25–35 years old [31]. Affected women suffer from symptomatic dysmenorrhea, abnormally high menstrual pain and bleeding. Aside for the caused pain endometriosis is also considered the main cause of infertility. A hormone treatment to block the growth of the tissue can be administered, but in long-term recurrent symptomatology and when the patient wants to become pregnant the disease is managed by means of classic endoscopic surgery [32], [33]. The number of reported cases of RMIS endometriosis surgery is very limited [34] and so, the use of robotic system for surgical removal of endometriosis is not currently recommended. Nezhat *et al.* [35] e.g., investigated 78 patients that were treated via RMIS during a year showing no particular benefit of robotic treatment for endometriosis outcome. The absence of haptic feedback in currently available RMIS is raised as a main drawback for robotic treatment of endometriosis.

### B. CO$_2$ Laser Endoscopy for Endometriosis

**Fig. 2.**
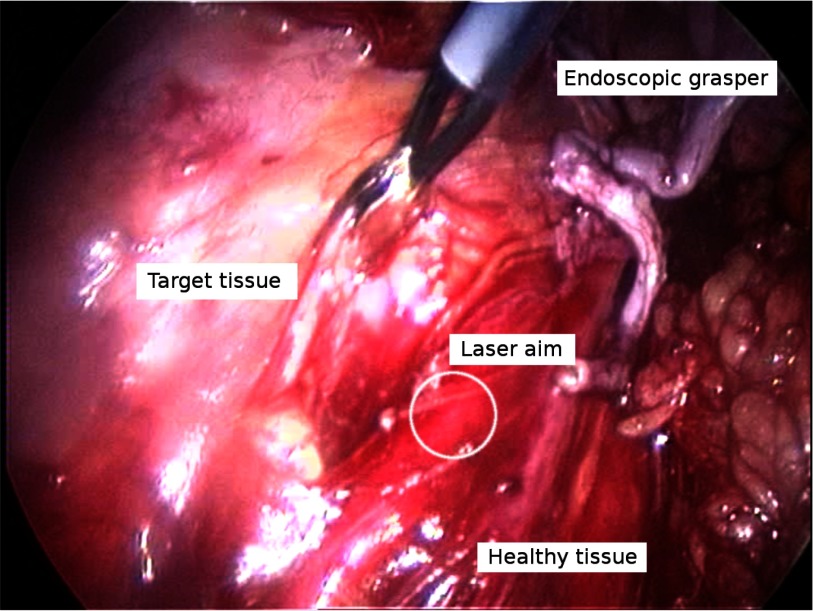
Endoscopic view of an endometriosis intervention; the different components that make up the scene are annotated.

Surgical endometriosis is considered to be one of the most challenging gynaecological interventions [36], requiring constant manipulation of the healthy peritoneum to isolate and separate it from endometriotic lesions. The challenge exists in removing all diseased tissue with minimal cutting margins, i.e., maximally preserving the surrounding healthy tissue (see Fig. 2). Since diseased and healthy tissue are closely interwoven, considerable skill is needed to entangle and separate these. Surgeons rely heavily on visual and haptic cues during this intervention.

For excision of tissue in delicate situations the use of CO$_2$ laser may be preferred over the use of bipolar scissors. The surgeon manipulates in one hand a grasper to grasp and stretch tissue and steers an ablation laser with his/her other hand. Good and stable coordination of both hands in this bimanual operation is crucial in order to prevent that tissue is unintentionally ruptured or wrongly ablated. In a typical surgical gesture where the endometriotic lesion is present on the surface of the associated organs (uterus, ovary or bowel) the surgeon starts by exposing the targeted endometriosis. He/she grasps and moves the surrounding tissue out of view, possibly fixing it in holding clamps. Next, the surgeon orients the tissue in front of the ablation laser. The tissue, recognizable by abnormal fibrosis, is then so to speak ‘peeled off’ with the laser. Due to excessive tension the tissue could get miss-aligned and the ‘cleavage’ plane (border between healthy and fibrotic tissue) could get lost. In such case there is a risk that healthy tissue is wrongly excised. Furthermore, large stress might tear the healthy tissue, even tear off the peritoneum or damage the organ.

After separation of the layers, the surgeon extracts the remaining of the lesion through the cannula and repeat the sequence until the last lesion is removed. Failing to fully remove the lesion could lead to regrowth of the endometriosis after the intervention which might then call for a new surgery.

### C. Model for Endometriosis RMIS

Currently there are no standard endometriosis training models. However, many different inexpensive models have been proposed in the past for exercising suturing or cutting tasks (orange [37], or clementine [38], foam [39], various fruits and vegetables [40], grapes [41]). All these and several other models have been investigated, but non were found to respond accurately to laser actions providing a repeatable mechanical and haptic behaviour. Hence, a model incorporating intrinsic difficulties of RMIS is proposed.

The model of the affected organ and endometriosis is represented by the pericarp of a tomato. The exocarp (skin) of the model, which is the stiffer part, takes the role of the fiobrotic tissue. The underlying mesocarp (flesh) corresponds then to the healthy organ tissue. In a raw tomato the mesocarp and the exocarp are strongly connected, in fact, stronger than is the case for a typical endometriotic lesion. The raw model makes it overly difficult to separate both. By precisely controlling the conditions of a warm bath the connectivity between layers can be adjusted. After thorough experiments it was found that a light red to red model of 200 g immersed during 2 min 20 sec in 400 ml of water at a temperature of ${\text{93}}^{\circ }\pm {\text{4}}^{\circ }$ provided acceptable realistic behaviour. Less heating leads to a stiffer connection; longer exposure weakens the connection too much, up to the point where both layers spontaneously separate. A quick and easy quality check is done by cutting and removing a small patch from the bottom of the tomato before the exercise to verify adequate peeling behaviour. Patient specific variations may be ‘programmed’ by adjusting heating time or employed temperature with this model. Also in real life such variation occurs. Typically it is determined by the level of fibrosis which on its turn is function of the age of the lesion.

### D. Peeling Exercise

**Fig. 3.**
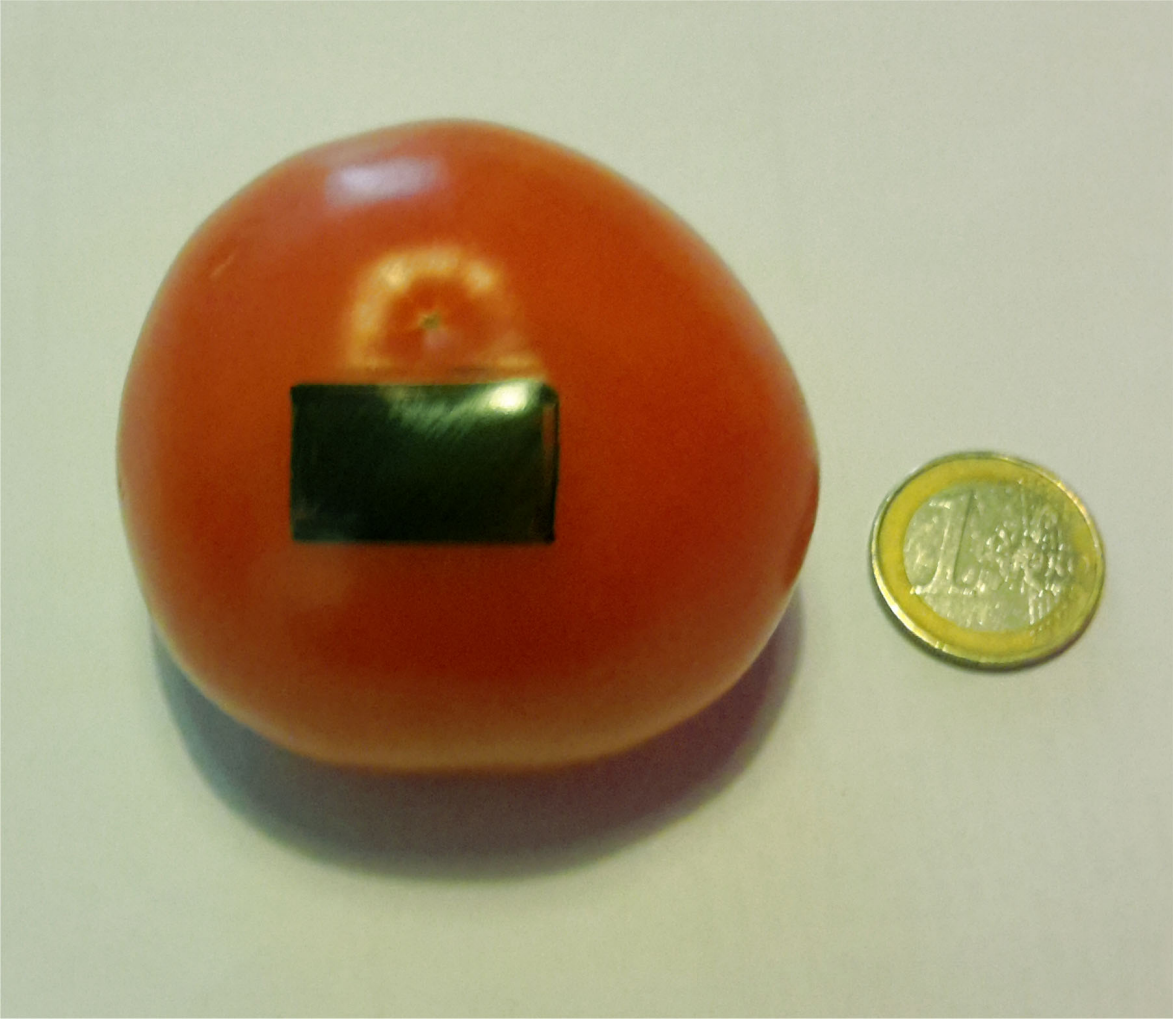
Photograph of tomato model marked with green pen; the part of the exocarp that needs to be ‘surgically’ removed from the mesocarp.

A robotic system and a procedural training exercise for RMIS have been designed using the abovementioned training model. A robotic platform - described in detail in next section – with two robotic arms, one holding a laser laparoscope and a second holding a grasper is used to remove a 21 mm × 13 mm rectangular patch of the exocarp. With a felt-tip marker the patch is coloured after the cooking (see Fig. 3) so that the participant has a clear view on the area to be removed. The participant is asked to remove the entire exocarp patch without leaving behind visual marks. In case such traces or marks remain, the exercise is classified as sub-complete. Instead, if the exercise is interrupted for some reason it is classified as incomplete. The user is asked to pay attention to limit the forces during manipulation and to ablate only targeted tissue, leaving the surrounding ‘healthy endometrium’ tissue intact. The envisioned exercise consists out of a sequence of four steps explained next.

*Approach:* First, the operator needs to position the instruments to obtain a proper visual overview and access to the site to be peeled. From a fixed start position the operator is to move the laparoscope over a distance of approximately 20 mm, then zoom in and focus the laser on the scene. The grasper travels over a similar distance. The scope is moved to keep the laser in view during this process. Interference between grasper and laser beam is to be avoided.

*Preparatory ablation step:* Next, the operator is to edge the perimeter of the marked patch with the ablation laser. The ablation should be sufficiently deep, piercing through the exocarp to support easy peeling afterwards. An indicative measure for proper cut through the exocarp is that the red color from the mesocarp starts appearing behind the blackened exocarp. If this level is not reached, the operator could unwillingly remove exocarp beyond the lesion including healthy tissue.

*Peeling and ablating:* After edging, the patch is to be peeled off. The peeling requires simultaneous and coordinated operation of both laser laparoscope and grasper. Initially, the grasper needs to protrude beneath the exocarp. By lifting the grasped patch, the ‘cleavage’ plane (plane separating healthy from diseased tissue) becomes visible. The laser is used then to progress the cleavage plane by ablating the connecting tissue between exo- and mesocarp. As the connecting tissue progressively loosens, the grasper must be continuously repositioned to maintain the tension on the exocarp and expose the new front of the cleavage plane. This step finishes when the targeted patch is physically separated from the mesocarp.

*Regrasping, peeling and ablating:* Occassionally the patch that is being processed might tear off. At that point the operator needs to re-grasp the remaining part of the patch to continue peeling and ablating until the whole patch is fully removed. When the operator is satisfied he/she is to specify this explicitly. At that time the exercise is considered finished.

During the entire exercise the operator can observe the motion of the instruments and of the endometriosis model on a monitor in front of him/her. The operator can be advised to work on certain movements and techniques. The instrument motion, exerted force, ablation commands and so on are all recorded. Performance metrics are calculated at the end of the exercise in order to help analysing the outcome of the exercise.

## III. Robotic Platform for Endometriosis RMIS

A bimanual bilateral robotic platform has been set up for RMIS endometriosis treatment. The different components of this platform are briefly introduced in Section III-A–III-E. Section III-F describes how the components are laid out for conducting the ESE experiments. Since the benefit of haptic feedback forms the subject of study, remote interaction forces are to be acquired and fed back to the operator. Section III-G explains how forces at the instrument tip are computed from a pair of external force sensors. Finally, Section III-H describes the bilateral controller that was implemented.

### A. System Overview

**Fig. 4.**
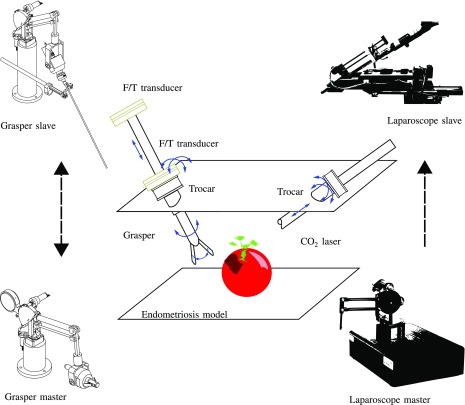
Experiment layout; an endometriosis model is placed remotely. The grasper is used to put the tissue under tension; the CO$_2$ laser is handled to remove a superficial patch while maximally keeping healthy tissue intact. The grasper is equipped with a force measurement system consisting of a pair of extra-corporeal force sensors. Both instruments access the region through a trocar. The laparoscope robot is controlled unilaterally while the grasper can be controlled either unilaterally or bilaterally.

The bimanual robotic platform is composed out of four main components, as presented in Fig. 4. At the slave side one robot is used to steer the laser laporoscope; a second robot controls a MIS grasper. At the master side two haptic joysticks are used to control the pair of slave robots. The surgeon holds these joysticks but also operates three foot-pedal buttons. These are used to control the ablation laser (on/off) or to clutch (decouple) each master-slave pair. This feature is essential as it allows independent re-positioning of master joystick's when boundaries of the master workspace are being reached. The slave side is equipped with commercial laparoscopic tools: a commercial laser laparoscope (Karl Storz GmbH & Co, Tuttlingen, Germany) interfaced with a digital camera (Richard Wolf GmbH, Knittligen, Germany). A 30 W Sharplan 30 C CO$_2$-laser (Esc Sharplan now Lumenis, Yokneam, Israel) and an halogen 250 W twin light source (K. Storz) are hooked up to this slave robot. The second robot is holding a modified surgical grasper (R. Wolf). The scissors handle are removed to be hold by the robotic system. This grasper is employed with a pair of force sensors to allow measuring the interaction forces between the grasper and the tissue it interacts with. The forces measured at the forceps are transmitted and rendered at the master console so that the operator can immediately feel the tension that is applied to the tissue. In order to restore the haptic link between the surgeon's hand and the instrument a bilateral controller is implemented. Finally, a monitor positioned at eye-level in front of the operator displays the endoscopic view as it is being captured by the laser laparoscope.

### B. Laser Laparoscopic Slave Robot

A first slave robot controls the position of the laser laparoscope. The laser, camera and light source are all integrated in the laser laparoscope. They are aligned parallel to each other and only separated by a small offset. The illumination and camera image will therefore move together with the laser. The laser will hence always be located centrally in the image. When the ablation is not activated a red mark is visible from a low-power Ne-He pointing laser. When the foot-pedal is pushed the pointing laser is switched with a high-power CO$_2$ ablation laser.

The Vesalius robot, an in-house developed robot featuring an ‘adjustable’ remote center of motion (aRCM) [42], is used to control the laser laparoscope. The robot possesses 2 DoFs to adjust the position of the RCM (hence aRCM) in order to align it with the incision into the patient. Once the RCM is positioned the robot can control the laparoscope with 3 DoFs: yaw, pitch and insertion/retraction $\boldsymbol {q}_{vesalius}=\left\lbrace \theta _{1}, \theta _{2}, d_{3}\right\rbrace ^{T}$. A rotation of the instrument about its axis is not foreseen as the tool (laser) is axi-symmetric. If needed, reorientation of the camera-image could be done digitally. When the RCM is properly aligned with the incision, the control is switched. The joints $\boldsymbol {q}_{vesalius}$ are then computed so that the laparoscope tip tracks the input commands of the master joystick in Cartesian space. A detailed description of the system can be found in the patent by Tang *et al.* [42]. The laparoscope is attached to the holder of the Vesalius robot. A light source, a Hopkins monocular telescope, and a Nezhat laparoscopic coupler (Lumenis) are included in the scope. The coupler connects the laparoscope to a 15 W CO$_2$ laser generator. The shutter of the laser beam is connected to a foot pedal switch. The latter has been modified to allow recording of the pedal state by the data acquisition system.

### C. Master Device Controling the Laser Laparoscope

A PHANToM Premium 1.5 (3D Systems, South Carolina, US) haptic display system [27] has been selected as input device to control the laser laparoscope. Its first 3 DoFs can be conveniently mapped to the DoFs of the laser robot. Being highly backdrivable, the PHANToM features low inertia and torque ripple, which makes it ideal to render clean - somewhat low amplitude (continuous forces up to 1.3 N) forces to the user. In theory these forces can be used to feed back interaction forces measured at the slave side. However, as the laser is not supposed to contact tissue, the full force range of the PHANToM can be used to compensate for gravity, to render artificial damping and to set virtual bounds, e.g., to indicate when workspace limits are reached (both for master and slave robot). The power-electronics and native software has been replaced to allow integration into the overall system developed with OROCOS [43] running on a platform under GNU/Linux Ubuntu 12.04 with RT Preempted kernel. The tip of the laser laparoscope follows the trajectory that the operator applies on the stylus of the PHANToM device. In theory these forces can be used to feed back interaction forces measured at the slave side. However, as the laser is not supposed to contact tissue, the full force range of the PHANToM can be used to compensate for gravity, to render artificial damping and to for put virtual bounds e.g., to indicate when workspace limits are reached (both for master and slave robot).

### D. Slave Robot for Controlling a Surgical Forceps

A slave robot was developed to control a Wolf 8393.911 laparoscopic grasper (R. Wolf). Operation of such grasper requires 4 motion DoFs (yaw, pitch, roll and insertion/retraction) and 1 additional DoF to open and close the grasper. The LoTESS, a 3 DoF in-house developed haptic device [28] served as base of this slave robot. Compared to the PHANToM, LoTESS has a much larger output force (continuous forces up to 12 N) which makes it much more suitable for the heavier grasping tasks. At the LoTESS’ end-effector an additional mechanism was built to rotate the forceps about the longitudinal axis and to open and close the forceps. The rotation is established by a DC motor Amax 2.5 Watt (Maxon Motors AG, Sachseln, Switzerland). A small pneumatic cylinder EG-PK (Festo AG, Esslingen am Neckar, Germany) mounted on the rotatory unit retracts to close the forceps. This mechanism replaces the native handle of the Wolf grasper. The setup is augmented with a mechanical passive RCM mechanism [44], a cannula that prevents excessive forces of the instruments on the entry port. Additionally, a pair of passive joints is inserted between the original LoTESS end-effector and the add-on forceps actuation system. The orientation of the grasper about its axis and the grasping state follow from the abovementioned extension to the LoTESS. To estimate the interaction force a method with 2 external force sensors, described by Willaert *et al.* [30], [44], is adopted and expanded. A first 6 DoF force/torque transducer Nano43 (ATI Industrial Automation, North Carolina, US) is positioned proximal to the pair of passive joints that were added between the LoTESS end-effector and the add-on forceps actuation system. A second Nano43 force/torque sensor is integrated into the passive RCM mechanism. The force that is applied on the tissue can then be estimated following the procedure explained in Section III-G. Overall, the result is a 5-DoF controllable grasper. The system has submillimeter positioning precision and delivers a continuous force of up to 7.5 N and a grasping force of 0.4 N. A torque of 0.425 Nm can be exerted about the instrument axis. Preliminary experiments showed that this specifications would suffice to finish successfully the task.

### E. Master Controling the Laparoscopic Grasper

Isomorphic master and slave robots are known to simplify bilateral control. For this reason a second LoTESS master robot was chosen to steer the slave robot that controls the surgical grasper. In prior work a very good bilateral coupling has been shown between both 3 DoF LoTESS systems [30], [44]. Here, just as the slave robot, the master robot has been expanded to a 5 DoF system, by adding a grasping-rotation module at its end-effector. A rotating unit was added and mounted at the end-effector of the LoTESS master. This module was hinged in a 2 DoF passive wrist to some extent replicating the configuration at the slave side. A handle for controlling the pinching of the grasper and the rotational motion of the instrument about its axis, is mounted at the level of the rotating unit. Thanks to the passive wrist, the user can comfortably handle the grasper and control the gripping force and orientation about the instrument axis. A miniature parallelogram that is driven by a pneumatic cylinder counteracting a return-spring is used to control the pinching motion. The result is a 5-DoF controllable haptic master with submillimeter positioning precision. that delivers a continuous force of up to 12 N, an gripping force of 0.45 N, and a torque of 0.128 Nm about the instrument's rotation axis.

### F. Platform and ESE Layout

The pair of master and slave robots, foot pedals and the laparoscopic view monitor are set up so that the user can work comfortably during long periods of endometriosis RMIS. The master robots are desktop devices. Their height is adjusted for easy access from an OR chair - with arm rests - that is positioned in front of a normal desk. The laser robot is positioned here at the right hand-side, while the forceps is operated by the left hand. For left-handed users both systems could be interchanged. Three foot pedal switches control the actions of the laser beam and the clutching mechanism of each pair of robots. Thanks to the clutching function the user can steer the slave robots over larger distances without reaching the end of the master's workspace. The operator can also choose at any time to clutch in order to position a master joystick in a more ergonomic position, e.g., to ensure operation under maximal positioning precision. At the patient side, the two slave robots are positioned at an angle of ${\text{90}}^{\circ }$ with respect to the ESE model. This configuration corresponds to the envisioned real-world situation. The model is enclosed in an aluminium frame covered with drapes that replicate the body wall, The drapes also prevent that reflected laser light departs from the ‘body’ and e.g., hits bystanders or participants to the experiments.

### G. Force Measurement

**Fig. 5.**
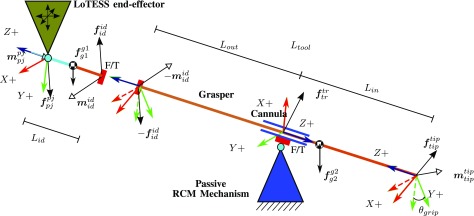
Free body diagram to derive the static equilibrium of the instrument driver and of the instrument itself. A first force transducer connects the two bodies. The instrument driver is hingedly connected via a 2 DoF passive joint to the end effector of the LoTESS. Rotation of the instrument about its axis is accomplished through the instrument driver. The instrument passes a cannula that is hinged in a 3 DoF passive joint. A second force transducer mounted at this point measures the interaction forces exerted on the body wall. All forces measured by the first force sensor that cannot be explained as originating from the body wall are thus caused by interaction with the targeted organ; here such interaction is assumed to take place at the tip of the instrument.

Force sensing is an essential component of a system that claims offering high-quality force feedback. In a medical situation foreseeing force sensing is not straightforward, since force sensors need to be compact, robust and sterile In this work, tip forces are calculated by means of two externally placed force sensors as described by Willaert *et al.* [30], [44]. As the approach by Willaert relies on assumption that the handle force sensor is at the wrist point, does not hold here, this method is expanded in the following. Fig. 5 provides a schematic free-body diagram of the components involved in force sensing. The instrument is collinear and rigidly connected to the ‘instrument driver’ - a motorized add-on designed to rotate the instrument about its own axis and to open and close the forceps.

This combination is hinged (left-hand side in Fig. 5) via a 2-DoF passive joint $ \left\lbrace pj \right\rbrace $ and a first force sensor to the end-effector of LoTESS. The force transducer forms the interface between the instrument and the instrument driver at the instrument driver point $ \left\lbrace id \right\rbrace $. The instrument further pivots and slides through a cannula attached to the passive wrist of the RCM mechanism. The coordinate frame $ \left\lbrace tr \right\rbrace $ coincides with the pivot point of the trocar/cannula. The second force transducer carries the trocar. A coordinate frame $ \left\lbrace tip \right\rbrace $ is further attached at the instrument's tip.

Wrenches $\boldsymbol{w} = \left[\boldsymbol{f}^{T}, \boldsymbol{m}^{T}\right]^{T}$ can also be expressed introducing different components and frames. From the free body diagram of the instrument the wrench generated by the connection at the instrument driver $ \left\lbrace id \right\rbrace $ can be calculated and expressed on $ \left\lbrace tip \right\rbrace $ as

\begin{align*}
\boldsymbol{w}^{tip}_{id} = {\left[\begin{array}{cc}\boldsymbol{R}^{tip}_{id} & \boldsymbol{0}_{3\times 3} \\
\boldsymbol{S}\left(\boldsymbol{r}^{tip}_{id}-\boldsymbol{r}^{tip}_{tip} \right)\boldsymbol{R}^{tip}_{id} & \boldsymbol{R}^{tip}_{id} \end{array}\right]} {\left[\begin{array}{c}-\boldsymbol{f}^{id}_{id} \\
-\boldsymbol{m}^{id}_{id} \end{array}\right]} \tag{1}
\end{align*}

where $\boldsymbol{f}^{id}_{id}$ and $\boldsymbol{m}^{id}_{id}$ are measured by the force sensor, $\boldsymbol{R}^{tip}_{id}$, $\boldsymbol{r}^{tip}_{id}$ and $\boldsymbol{r}^{tip}_{tip}$ are the rotation and position of corresponding frames, calculated from the forward kinematics and $\boldsymbol{S}(\cdot)$ is the cross product operator expressed as a $3\times 3$ skew-symmetric matrix. One can similarly describe the wrenches generated at the connection of the trocar $ \left\lbrace tr \right\rbrace $, calculated and expressed in the $ \left\lbrace tip \right\rbrace $ frame to be

\begin{align*}
\boldsymbol{w}^{tip}_{tr} = \left[ {\begin{array}{c}-\boldsymbol{R}^{tip}_{tr} \boldsymbol{f}^{tr}_{tr} \\
-\boldsymbol{S}\left(\boldsymbol{r}^{tip}_{tr}-\boldsymbol{r}^{tip}_{tip} \right)\boldsymbol{R}^{tip}_{tr} \boldsymbol{f}^{tr}_{tr} \end{array}} \right] \tag{2}
\end{align*}

where $\boldsymbol{f}^{tr}_{tr}$ and $\boldsymbol{m}^{tr}_{tr}$ are measured by the force sensor, and $\boldsymbol{R}^{tip}_{tr}$ and $\boldsymbol{r}^{tip}_{tr}$ are calculated from the forward kinematics. Since accelerations are considered low, a static analysis is adopted here. In such case the reaction force at the tip of the instrument becomes:

\begin{align*}
\boldsymbol{w}^{tip}_{tip} = -\boldsymbol{w}^{tip}_{id} -\boldsymbol{w}^{tip}_{tr} -\left[{\begin{array}{c}\boldsymbol{g}^{tip}_{g} \\
\boldsymbol{0}_{3\times 1} \end{array}}\right] \tag{3}
\end{align*}

One can see the gravity $\boldsymbol{g}^{tip}_{g}$ appearing in (3), however this force is more or less constant. In practice this force component is eliminated by resetting (zeroing) the force sensor before each experiment.

### H. Control Strategy

In this subsection the control of the pair of instruments is described. A distinction is being made between the control of the laser laparoscope and that of the grasping forceps. Since the laser is not supposed to touch the organs, force does not need to be fed back here, hence a unilateral controller suffices. For the grasping forceps both a unilateral and a bilateral controller have been implemented. The effect of haptic feedback is investigated by comparing the difference in performance between the two controllers for the grasping forceps. In the following some extra information is provided w.r.t. these controllers.

#### 1) Unilateral Control of the Laser Laparoscope Robot

A unilateral controller is set up between the PHANToM Premium 1.5 and the Vesalius laser laparoscopic robot. Positions of PHANToM's end-effector are tracked by the tip of laser laparoscope. As long as the required speed and acceleration are not too large the stepper motors manage to track the requested trajectory. Note that damping is injected at the master side to keep velocities low. The open-loop position controller at the slave side behaves therefore stable during all our experiments. Damping at the master side enhances the overall positioning precision. At the same time it also lowers the chance that the laser laparoscope would lag and fail to follow the operator's input commands. As a discrepancy exists between workspaces, an additional restriction is applied on the master to ensure that its position stays within a cone of 50° aperture and 120 mm length which corresponds to the foreseen slave robot workspace. Such restriction avoids discontinuous behaviour e.g., when the master robot would attempt to steer the slave beyond its limits. As there exists a scale factor $\lambda$ between master and slave the cones at master and slave side will be equally scaled. The user may wish to clutch (decouple) the master from slave, e.g., to take on a more ergonomic position. Then the cone at the master side is shifted at such occasion in order to keep the correspondence. Thus the relative pose of the master w.r.t. the cone at the master side is not altered by clutching. To formalize this behaviour the function $f_{cone}(\cdot)$, which simply restrains a position $\boldsymbol{x}$ to stay within a cone $C_{tr}$, is defined as:

\begin{align*}
f_{cone}(\boldsymbol{x}) = \mathop{\text{arg}\,\text{min}}\limits_{\boldsymbol{x}_{c}} \Vert \boldsymbol{x}_{c} -\boldsymbol{x} \Vert \mid \forall \boldsymbol{x}_{c} \in C_{tr}. \tag{4}
\end{align*}

The apex of the cone maps to the slave's remote-center-of-motion central in the slave's workspace. Without loss of generality the cone axis is aligned vertically in this work. Furthermore, singularities are handled by restricting motions to lie on the cone axis, the projection $x_{c}$ of $x$ on that axis, when reaching the apex sufficiently close - at a distance below a predefined threshold. Borrowing from Virtual Reality haptic rendering techniques a god-object [45] (also called proxy [46]) is used. This virtual representation of the actual position of the robot stops when a virtual collision happens generating a force pulling from the device end-effector. The god-object $\boldsymbol{x}_{go}$ is the representation of the master position $\boldsymbol{x}_m$ restricted to $C_{tr}$ and obtained as

\begin{align*}
\boldsymbol{x}_{go} = f_{cone}(\boldsymbol{x}_{m}). \tag{5}
\end{align*}

The position of the god-object is then send to the slave robot to track.

The control law at the master side that captures above features can be written as:

\begin{align*}
\boldsymbol{F}_{m} = K_{p}(\boldsymbol{x}_{m}-\boldsymbol{x}_{go})-B_{v}s\boldsymbol{x}_{go} + \boldsymbol{G}(\vphantom{q}{\boldsymbol{q}}\vphantom{q}_{m}) \tag{6}
\end{align*}

where $K_{p}$ is the proportional gain, $B_{v}$ is the damping term. $G(\vphantom{q}{\boldsymbol{q}}\vphantom{q})$ is used to compensate for gravity at a given configuration. Because of the implemented workspace constraint $f_{cone}(\cdot)$ the controller returns a zero proportional term when the virtual proxy and the master tip coincides inside the cone. In such case only damping and gravity compensation are active. A virtual spring can be perceived as soon as the operator exceeds the virtual cone.

At the slave side the motor action is calculated as difference between the slave position $\boldsymbol{x}_{s}$ and the god-object position. A simple PD-type (proportional-derivative) closed-loop position controller is then implemented.

\begin{align*}
F_{s} = \boldsymbol{C}_{s}\cdot \left(\boldsymbol{x}_{go}-1/\mu \boldsymbol{x}_{s}\right) +G(\vphantom{q}{\boldsymbol{q}}\vphantom{q}_{s}) \tag{7}
\end{align*}

#### 2) Unilateral and Bilateral Control of the Forceps

The forceps can be controlled unilaterally or bilaterally. In the former case the same control scheme as explained above is adopted. In the latter case, the interaction force is measured according to the setup described in Section III-G. Based on this force a three channel control strategy **P-P-F$_{e}$** is implemented. This controller is a particular implementation of the more general four channel controller from Lawrence *et al.* [47]. In concrete terms, the forwarded forces generated at the master side are *not* forwarded for closed loop control at the slave side. The forward control law is the same (7) as for the unilateral controller. For the backward control law, both the scaled position (scale factor $\mu$) and the scaled force (scale factor $\lambda$) are fed back to the master:

\begin{align*}
F_{m} = C_{m}\cdot \left(\boldsymbol{x}_{s}-\mu \boldsymbol{x}_{m}\right) -\lambda \cdot \boldsymbol{F}_{e} + G(\vphantom{q}{\boldsymbol{q}}\vphantom{q}_{m}) \tag{8}
\end{align*}

where $F_{e}$ is the environment reaction tip force and which is calculated from (3) as in $F_{e}=-\boldsymbol{R}^{m}_{tip}\boldsymbol{f}^{tip}_{tip}$. Gains were found experimentally. During the tuning process we strived to maximise system transparency but gave higher priority to system stability. This lead to fairly low gains for $C$ and $G$ components and inevitably a certain tracking error (see Fig. 7), but allowed us to avoid any instabilities during the intervention, including high-frequency content impacts such as collisions with rigid environment.

### I. Auditory Feedback

It was decided after some early preliminary experiments to introduce a high pitch auditory cue as some participants - especially in the unilateral case - would not care too much about applying excessively large forces. So the acoustic feedback served as a reminder that exerted forces should be kept low.

A monotone auditory signal was given after surpassing a force level of 1.75 N. The force threshold was chosen from the initial experiments so that highly skilled operators could conduct a successful completion of the exercise without rising the alarm. Since the acoustic feedback was binary, the operator could not deduce the force magnitude from this feedback. In the discussion part of this paper the effect of this cue on the performance and user experience is investigated.

## IV. Experiments

In order to evaluate the impact of the use of haptics in a complex robotic surgical procedure a user campaign has been organised. Subjects were invited to execute the ESE, described in Section II, three times under two conditions in random order. The slave robot was operated either under unilateral control, i.e., without haptics feedback, or under bilateral control, i.e., with haptic feedback. In a first round, 10 mechanical and biomechanical engineers working at the institute took part in a pilot test, providing preliminary data and feedback regarding the protocol. These pilot results were used to optimize the protocol, the number of conditions to be tested and the scheduling. In a second round 9 surgeons of varying levels of expertise participated in the experiments.

### A. Training on the Robot Platform

Every subject received a training to get acquainted with the system before starting the first experiments. Subjects were first explained (where necessary) the workflow of endometriosis and the relation to the ESE experiment that has been designed. The objective of the task was explained. Subjects were taught how to command the slave robots through the pair of master joysticks. Next, a demonstration was given showing how to execute the simulated surgical task. Subsequently the subjects were given the opportunity to practice on their own. The participants were free to ask anything that was unclear during this exercise session. Participants got equal opportunity to train both operation modes. Once they indicated to feel comfortable with the platform and the exercise (the whole training process took about 30–40 minutes), the experiments and recordings started. In order to avoid the learning curve effect the experiments were randomized.

### B. Population

Nine surgeons with different specialities ‘obstetrics and gynaecology’ (7), ‘ENT surgery’ (1) and ‘pediatric surgery’ (1) participated to the experiments. The level of experience varied among participants. Some described themselves as early residents others were licensed surgeons. The age of participants ranged from 26 to 39 years. Five of the participants were female, four were male. One female surgeon possessed regular experience with the da Vinci robotic system on other gynaecologic interventions. A second female surgeon indicated to have had considerable experience with the da Vinci's training platform. All the male participants had at least received a training session with the da Vinci robotic system. One male surgeon had actually performed robotic interventions in the past. Further, 2 participants indicated to play videogames, whereas another surgeon plays musical instruments. About 44% of the participants indicated to conduct laparoscopic interventions under the supervision of a senior.

### C. Subjective Questionnaire

After the last experiment surgeons were invited to participate to a questionnaire to gauge their experience and opinion about the conducted experiment. Table I summarizes the results of the questionnaire. Participants had to score between 1 (not agree) and 5 (fully agree). The P-value was calculated with Kruskal-Wallis to investigate the correlation. The data was grouped by level of experience of the participant and by experience with robots. If data belonged to the same distribution, then opinions are consistent.

**TABLE I:** Analysis of the Answers to the Subjective Questionnaire Provided After Completing the Exercise Session. P-value Corresponds to the Level of Correlation of a Given Question.

Question: Score the sentence (1–5); 1: Disagree, 5: Fully agree	Grouped by education				Grouped by robot exp.				P-value
	Resident		Licensed		Non exp.		Experienced		
	Mean	Std	Mean	Std	Mean	Std	Mean	Std	
I got tired after the tests	2.5	1.0	2.3	1.2	3.0	1.0	2.2	1.0	0.77
I got stressed during the tests	2.3	1.5	1.3	0.6	2.7	2.1	1.7	0.8	0.64
The task was difficult	2.7	1.0	2.7	1.2	3.0	1.0	2.5	1.0	0.94
The task was representative of endometriosis laser surgery	3.5	0.5	4.0	0.0	3.3	0.6	3.8	0.4	0.33
The laser robot was tiring	1.3	0.5	1.3	0.6	1.3	0.6	1.3	0.5	$>$0,99
The grasper robot was tiring	3.0	1.4	1.7	0.6	2.0	1.0	2.8	1.5	0.43
The use of the laser robot intuitive	4.0	0.6	4.0	0.0	3.7	0.6	4.2	0.4	0.77
The use of the grasper robot intuitive	2.5	0.8	3.3	0.6	3.0	1.0	2.7	0.8	0.50
The haptic controller …									
…helped me to control the force	3.7	1.5	4.3	0.6	5.0	0.0	3.3	1.2	0.06
…acted as a barrier preventing me from smashing the surface	3.8	1.6	4.3	0.6	5.0	0.0	3.5	1.4	0.22
…made me slower	2.7	1.5	2.7	0.6	3.0	0.0	2.5	1.5	0.88
…made me more careful	4.0	0.6	4.0	1.0	4.7	0.6	3.7	0.5	0.28
…made me more accurate	3.5	1.4	4.3	0.6	4.3	1.2	3.5	1.2	0.53
The auditory cue for force was useful for me	3.0	1.7	3.7	1.5	4.3	0.6	2.7	1.6	0.46
I was more focus on the auditory cue than the haptics	2.0	0.9	1.7	0.6	2.0	0.0	1.8	1.0	0.91
I feel more confident with haptics than with non-haptics	3.7	1.5	4.0	1.0	4.0	1.0	3.7	1.5	1.00
I would prefer haptics if I operate with the robot	4.0	1.1	4.0	1.0	4.0	1.0	4.0	1.1	$>$0,99
$\divideontimes$ Regarding content validity									
Practicing more with …									
…DaVinci would help me on this exercise	3.8	1.1	4.3	0.6	4.5	0.7	3.8	1.0	0.70
…an endoscope in OR would help me on this exercise	2.8	0.8	3.7	0.6	3.5	0.7	3.0	0.9	0.49
…an endoscope in another training would help me on this exercise	3.2	1.1	3.7	0.6	4.0	0.0	3.2	1.0	0.72
…a grasper in OR would help me on this exercise	3.4	0.9	3.7	0.6	4.0	0.0	3.3	0.8	0.78
…a grasper in another training would help me on this exercise	3.2	0.8	3.7	0.6	4.0	0.0	3.2	0.8	0.48
The exercise could be applied for manual interventions	2.5	1.0	4.0	1.0	3.5	0.7	3.0	1.4	0.28
I would recommend this exercise to …									
…understand an endometriosis surgical intervention	3.4	1.3	4.3	0.6	4.0	0.0	3.7	1.4	0.86
…improve my general laparoscopic skills	3.8	0.8	3.0	0.0	3.0	0.0	3.7	0.8	0.29
…improve my robotic laparoscopic skills	3.8	0.8	4.7	0.6	4.5	0.7	4.0	0.9	0.49
…practice CO$_2$ lasering skills	4.0	0.7	4.0	1.0	4.0	0.0	4.0	0.9	$>$0,99
$\divideontimes$ Regarding face validity									
Does the exercise look like endometriosis surgery	3.0	0.7	4.0	0.0	3.5	0.7	3.2	0.8	0.48
The model could be used also for manual interventions training	3.5	0.6	4.5	0.7	4.0	0.0	3.8	1.0	0.51
The interaction between grasper and tissue seemed realistic	2.0	1.0	3.0	1.4	2.5	0.7	2.2	1.3	0.51
The tissue excision behaviour was realistic	2.6	1.1	3.5	0.7	3.0	0.0	2.8	1.3	0.84
The endoscopic view was like looking at the monitor on the OR	4.0	0.7	3.5	0.7	3.5	0.7	4.0	0.7	0.70
The workspace, volume/space used, was adequate	3.4	0.5	3.5	0.7	3.0	0.0	3.6	0.5	0.73

### D. Data-Gathering During the Experiments

An advantage of using robotic systems for training over e.g., box trainers is that full access to all sensory data is available. It thus is possible to record and analyse how instruments are moving and interacting. External tracking systems [48] or special image processing techniques [49] are not needed as position and forces are directly measured.

The following parameters were recorded: experiment identifier, subject gender, subject age, subject preferred hand, type of controller (with/without haptic feedback) and order of the experiment.

The following signals were recorded at 60 Hz: *1)* configuration of the endoscopic master $\vphantom{q}{\boldsymbol{q}}\vphantom{q}_{em}$, *2)* configuration of the endoscopic slave $\vphantom{q}{\boldsymbol{q}}\vphantom{q}_{es}$, *3)* configuration of grasper master $\vphantom{q}{\boldsymbol{q}}\vphantom{q}_{gm}$, *4)* configuration of grasper slave $\vphantom{q}{\boldsymbol{q}}\vphantom{q}_{gs}$, *5)* status of the foot pedals $\boldsymbol{S} = \left[s_{g}, s_{e}, s_{l}\right]^{T}$, *6)* forces at the trocar $\boldsymbol{w}_{tr}$, *7)* forces at the grasper handler $\boldsymbol{w}_{h}$, and *8)* forces at the grasper tip $\boldsymbol{w}_{tip}$.

**Fig. 6.**
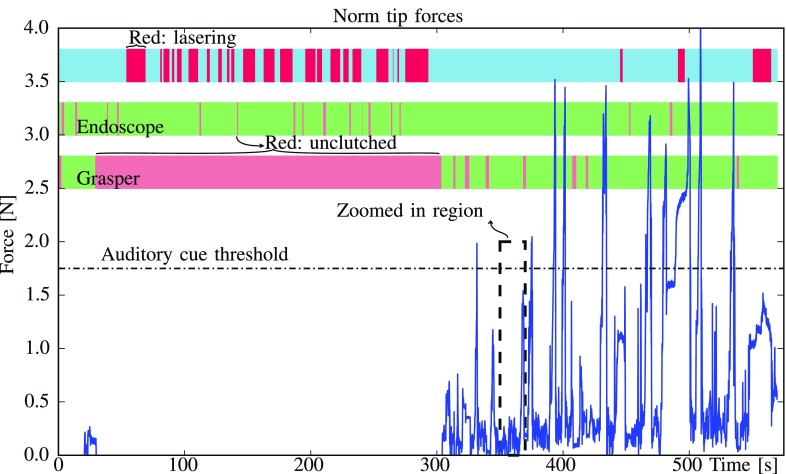
Time series of $|\boldsymbol{f}_{tip}|$ through one exemplary exercise in which haptic feedback was available. Coloured bars on top indicate the activation of the CO$_2$ laser and the ‘clutching’ state of the laser and the grasper. During the first stage of the exercise, contact forces are close to zero as the surgeon is only using the laser for edging and the grasper is unclutched; in the second phase the forceps is actively used to position the patch for the laser.

**Fig. 7.**
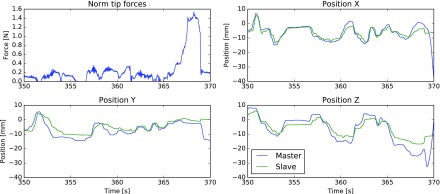
Zoom in on the $|\boldsymbol{f}_{tip}|$ time series of the region marked in Fig. 6. Next, tracking performance of the slave grasper and the respective master – over the same region. Note that no ‘clutching’ took place during this time window.

An example of the evolution of the tip force over time under bilateral control is drawn in Fig. 6. The status of the foot pedals is depicted alongside. Fig. 7 zooms in on an interesting area between 350 and 370 s. The tracking performance of the grasper with respect to the master trajectory for the same time interval is depicted in alongside. Main discrepancies between both trajectories are the result of the controller stiffness using for the teleoperation.

**Fig. 8.**
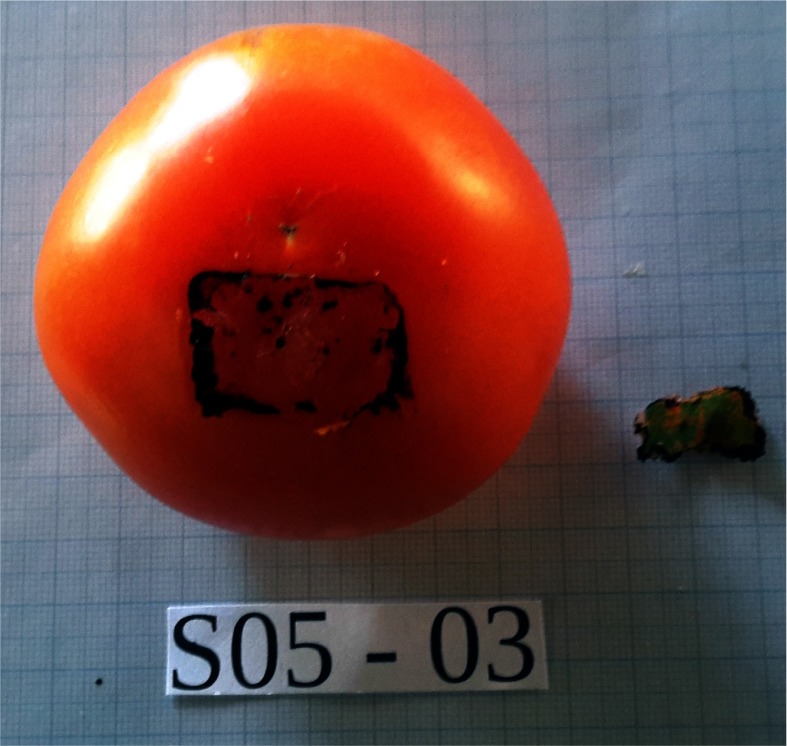
Sample of processed model of 3rd exercise of surgeon number 5. A single exocarp patch visible at the right. Good outcome visible as no green coloured mark, targeted tissue, remains on the surface of the tomato.

After each exercise, a digital photograph of the model was taken as shown in Fig. 8. Two videos were taken over the entire duration of the exercise. One video captures the endoscopic view as seen by a camera attached to the endoscope. A second video is taken from a side perspective of the model. It captures the surroundings and the motion of the slave robots.

### E. Metrics for Performance Analysis

Laparoscopic psychomotor skills can be assessed by different metrics computed form recorded time series [50]–[52]. Here, all acquired data was post-processed to compute metrics that tell something about the applied forces, the paths that were followed by the grasper, instants and duration of laser and clutching actions. Also the smoothness of the motion or uncontrolled collisions that took place are gauged. Such collisions are counted when the tools, laparoscope and grasper, make contact out of view with such a force that the acoustic cue is risen or when the grasper pierces the surface at an unwanted location and the user retracts subsequently. Table II summarizes the results of a statistical analysis that was conducted on the calculated metrics. In order to check the validity of the assumptions required for some statistical tests, a D’Agostino and Pearson normality omnibus test was executed. The hypothesis of having datasets coming from a normal distribution can be rejected in most cases based on skewness and kurtosis of the distribution. The datasets often had a bimodal shape. As a result, non-parametric Mann-Whitney tests were run. The P-values were computed for rejection of the hypothesis that the ranks of the groups (haptics and non-haptics) are equally distributed. The algorithms of the R libraries were used here.

**TABLE II:** Summary of the Metrics Extracted for Every Exercise. Analysis is Grouped by Type of Controller Used (Unilateral Versus Bilateral). P-values From Running a Mann-Whitney Test Between the Groups are Indicated.

Description	Unit	Formula	Non haptics		Haptics		P-value
			Mean	SD	Mean	SD	
Maximum force measured at the tip	$N$	$max\left(\Vert \boldsymbol{f}_{tip}\Vert \right)$	6.87	4.00	4.28	1.96	0.0019
Mean force measured at the tip	$N$	$mean\left(\Vert \boldsymbol{f}_{tip}\Vert \right)$	0.81	0.42	0.48	0.30	0.0023
Overall execution time	$s$	$\int _{0}^{T}dt$	577	227	615	330	0.89
Total time that the laser was active	$s$	—	175	57	201	87	0.34
Total time that the auditory cue was given	$s$	—	85.75	71.07	55.45	91.75	0.0011
Travelled path by grasper master	$mm$	$\int _{0}^{T}{ds}$	5333	4970	4590	3353	0.61
Number of pieces of tissue being peeled (fewer is better)	—	—	1.19	0.48	1.08	0.28	0.58
Number of times …							
…the user had an accident with the instruments	—	—	1.04	1.02	0.36	0.64	0.0077
…the laser pedal was pressed	—	—	23.2	8.6	25.4	13.0	0.80
…the user fully closed the grasper	—	—	18.6	13.7	24.8	24.1	0.55
…the grasper was unclutched (off)	—	—	4.22	4.37	3.88	4.87	0.60
…the endoscope was unclutched (off)	—	—	16.6	10.0	17.8	12.6	0.73
…the auditory cue was given	—	—	63	50	42	42	0.016
Time that the grasper was unclutched	$s$	—	67.0	97.2	73.3	115.8	0.66
Time that the endoscope was unclutched	$s$	—	19.3	8.98	22.1	16.0	0.96
Jerkiness of grasper master	$mm^{2}/s^{6}$	$j(\boldsymbol{r}_{gm})$ $^{\rm a}$	40.6	31.6	32.7	19.4	0.29
Jerkiness of endoscope master	$mm^{2}/s^{6}$	$j(\boldsymbol{r}_{em})$	3.82	3.71	3.22	2.31	0.86
Jerkiness of grasper slave	$mm^{2}/s^{6}$	$j(\boldsymbol{r}_{tip})$	34.4	34.9	21.2	18.4	0.036
Jerkiness of endoscope slave	$mm^{2}/s^{6}$	$j(\boldsymbol{r}_{es})$	0.03	0.02	0.03	0.02	$>$0.99

^a^ $j(\boldsymbol{r})=\frac{1}{2T}\int _{0}^{T}{\left(\delta ^{3}\boldsymbol{r}/\delta t^{3}\right)^{2} dt}$

**Fig. 9.**
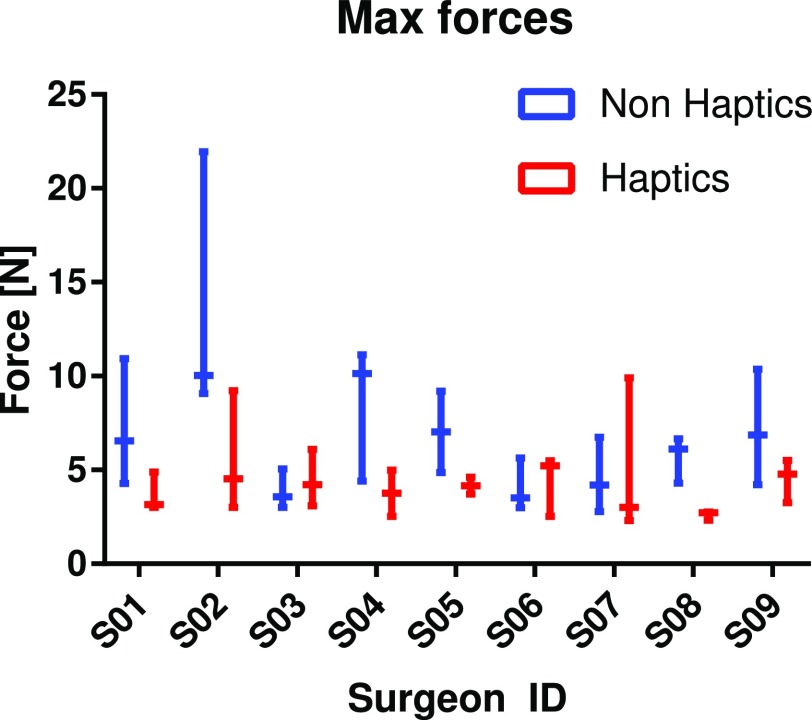
Analysis of the metric “maximum force” grouped by surgeon and classified by type of controller.

**Fig. 10.**
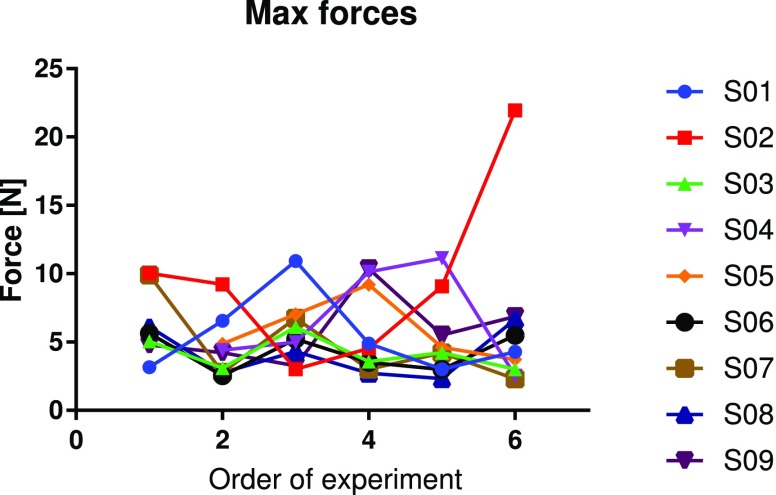
Analysis of the metric “maximum force” represented individually for every exercise in chronological order of execution and matched by surgeon.

Fig. 9 analyses the primary outcome: the maximum force. The data is grouped by condition and surgeon. The evolution of this metric along with the repetitions of the exercise is presented in Fig. 10. Note that all the data was included in our analysis. No points were rejected as outliers as the variability in background of participants was considered responsible for variations in performance. Nevertheless, all analyses were checked with outlier removal as well which resulted in more outspoken conclusions than those presented here.

## V. Discussion

When asked the surgeons about the validity of ESE, correlation was found among their most of answers. There was a very strong consensus (P $>$ 0.99) amongst surgeons, showing preference to have haptics to operate on this model (4.0). The surgeons displayed a similar consensus when indicating disagreement with the statement that use of the laparoscope robot was tiring (1.3). Questions regarding the content validity showed a significant correlation (P = 0.48–0.99) assigning high scores (3.12–4.12) in favour of using ESE to train other exercises such as CO$_2$ lasering or robotic laparoscopy. For example, surgeons agreed that the ESE exercise could be recommended to practice CO$_2$ lasering skills (4.0) or found ESE representative of endometriosis laser surgery (3.7). The relation between ESE and other exercises that address the same set of skills supports the content validity of ESE [53]. The weakest correlation (P = 0.28), yet agreeing (3.1), is found in using ESE to train manual interventions. A discrepancy was found where licensed surgeons were more convinced (4.0) to promote ESE for such training than novices.

Regarding face validity, it is interesting to see that while residents were ambiguous (3.0), licensed surgeons were agreeing that ESE looks like endometriosis surgery (4.0). Surgeons indicated with a correlation of P = 0.70 that the setup and the way the endoscopic view was presented on the monitor resembled the OR situation well (3.8). These scores confirm face validity of some aspects of the ESE. Surgeons were, on the contrary, less convinced about the realism of the interaction between tissue and grasper (2.3) and the behaviour of the excision (2.9) with correlations of P = 0.51 and P = 0.84 respectively. As the surgeons couldn’t maintain a large grasper closure force, the skin some times slipped off the forceps. Moreover, some surgeons point out that when dealing with older lesions, parts with fibrotic tissue would feel stiffer. The exocarp surface, instead, shows the same stiffness over its entire surface. A method where locally more or less heat is applied to program the local stiffness, or any other means, to create a varying higher stiffness, could be investigated to improve the realism of the model in further work.

Concerning the robotic platform, the laparoscope was found more intuitive to operate (4.0) than the grasper (2.7), a reasonable statement since the grasper is a more complex tool. The current manual laparoscopy is conducted without stereo-vision. However, surgical robotic devices such as DaVinci are known to offer excellent depth perception. A further study, whereby stereo-vision is offered as well, would be useful, providing a better understanding of the role of such stereo and how it outweighs haptic feedback. The closure DoF of the grasper tip was not always functioning at wish for it sometimes failed to immediately follow e.g., the closing command from the surgeon. This behaviour forced the surgeons to repeat this gesture. Some surgeons also expressed their preference to close the grasper more strongly. The current implementation was perceived to be less effective than that of traditional manual grasper, where a firm closed configuration can be maintained more easily. The small tracking error, result of the soft controller gains, was unnoticed. In future work a passivity observer passivity controller (PO/PC) [54] could allow installing less conservative controller gains and boost transparency without risking stability issues.

Encouraged by the validation of the ESE, the performance metrics are discussed in the following. The ‘peak’ and ‘average’ force per trial form the primary performance metrics. Table II shows a clear reduction of peak force (P = 0.002) when using the haptic controller. Peaks in force were occurring on diverse occassions, mainly, when the grasper collided with the model but also often when the tip impacted the laparoscope or other surfaces such as the floor. The average force is another primary outcome of this experiment. Forces are similarly reduced on average from 0.81 N down to 0.48 N under haptic feedback. If novice and licensed surgeons are analysed separately, the differences (between non-haptic and haptic execution) in peak and average forces are more outspoken for novices (P = 0.0014) and (P = 0.032) respectively, compared to licensed (P = 0.37) and (P = 0.09). When only looking at unilateral (non-haptic) controllers, licensed surgeons tend to show better values than novices on the primary metrics and other secondary metrics such as overall execution time or number of collisions. However the difference is not sufficiently statistically significant to confirm this tendency. This difference fades out when haptics is present.

As mentioned in Section III-I auditory cues were introduced to keep operators vigilant not to apply excessively large forces. Such auditory cues might have blurred to some extent the effect of pure haptic feedback as without it differences might be even more outspoken, since the cues were more present without haptic feedback (P = 0.001). Surgeons agreed (strongly P = 0.91) when marking not to focus on the acoustic signal (“The auditory cue was useful to me”, scored 1.7–2.0, Table I). Surgeons indicated that the cue was similar in pitch to cues that are already present in the OR today and that for this reason the cue might have gone a bit unnoticed. Haptic feedback, on the other hand, displayed a clear impact on the manipulation of the grasper. Participants strongly emphasized that haptic feedback felt as barrier that prevented them from piercing the surface (4.0). Participants agreed that haptic feedback helps the surgeon to control the force (3.9); they also indicated that haptic feedback makes them more careful (4.0). Uncontrolled collisions were also more prominent when haptics was not present (P = 0.0077). Note that uncontrolled collisions were counted by careful observation of the researcher guiding the experiment. In this sense it is a somewhat subjective measure and should be treated with care. In future work one could directly estimate the collisions by measuring contacts e.g., as changes in the electrical resistance between instruments. Typically collisions go unnoticed by the forward looking camera. A similar behaviour is found when looking at the amount of times the force exceeds 1.75 N which happens more often (P = 0.016) with a unilateral controller (63 times) compared to a haptic controller (42 times). Collisions lasted longer without force feedback as it took more time to notice and users found it more difficult to know in which direction to move in order to resolve the collision. With the haptic controller the feedback forces urge the operator to move the joystick in the direction opposite to the contact force, guiding the user thus to a safe pose. The bilateral controller adds extra inertia which slows down the motions. So overall, the improved performance observed by using the bilateral controller can be attributed to a combination of larger inertia and feedback of interaction forces. Further research is needed to determine which is the dominant contribution. Intuitively one would expect that increasing inertia would lead to larger impacts. Some out-of-view contacts also took place between the side of the tomato model or the floor. In one non-haptic experiment, the surgeon applied so much force to the side of the tomato model, that the straps, used to restrain the model on the table broke and the tomato feel out of the training box.

The time to completion is another important metric. While some argued in the past that haptics leads to a more efficient and a faster execution, others reported longer execution times. The experiments described in this work took longer with haptics enabled, although a significant difference was not present (P = 0.89). With the chosen **P-P-F$_{e}$** controller for haptic feedback, the inertia of the robot is felt. In a sense this might also have slowed down the execution. Regarding total execution time, one can notice that the standard deviation of the metric, about half of the mean execution time, is relatively high. Its variation is subjected to many factors and events that occur during each individual exercise. For instance, some participants, even after training, kept asking questions regarding the manipulation and strategy, finding more trouble in some exercises than others, typically such trials were much slower in execution. One aspect that is to be mentioned is that despite the effort to have reproducible test models, some variation among test samples was inevitable. Some models were softer than others to some extent simplifying the exercise. A comparison of performance between experiments could clarify this. However, as this factor was random it is believed that these moderate variations did not affect the results of this study.

## VI. Conclusion

This work investigated the impact of haptic feedback on the execution of complex bimanual robot-assisted lasering tasks. The Endometriosis Surgery Exercise (ESE) has been designed to replicate the delicate tissue manipulation characteristic of endometriosis treatment.

A bimanual telesurgical setup with unilateral and bilateral controllers were developed. Nine surgeons were asked to execute bimanual laser tasks with both unilateral and bilateral controllers. Surgeons indicated that the telesurgical system was intuitive to use. They also indicated that the designed ESE is formed challenging exercise that covers the most critical parts of endometriosis treatment. The experiments supported the content validity of ESE. The skills necessary to complete ESE were said to correspond to those needed to succesfully accomplish a real intervention. Whereas experienced robotic surgeons tend to argue that loss of haptic feedback is easily overcome by other senses (mainly vision), this work shows that haptic feedback could still make a difference when complex surgical interventions are concerned. The conducted experiments showed that thanks to the bilateral controller, the average and peak interaction forces reduced. The number of uncontrolled collisions also dropped The overall execution was longer, yet the difference was not significant. Surgeons preferred haptic feedback for executing ESE, even for surgeons with previous experience with commercial robotic systems.

Although a significant effort was put in the design of the telesurgical setup and ESE, further improvement is advisable. An optimization of the controllers, now running with moderate gain values, could increase the overall transparency of the system. At the same time, we remain interested to investigate in how far such optimization relates to improved outcome. By conducting a thorough analysis of the displayed performance levels we wish to come to this understanding.
